# ﻿Designation of the neotype of *Triatomadimidiata* (Latreille, 1811) (Hemiptera, Reduviidae, Triatominae), with full integrated redescription including mitogenome and nuclear ITS-2 sequences

**DOI:** 10.3897/zookeys.1076.72835

**Published:** 2021-12-08

**Authors:** Silvia Andrade Justi, Carolina Dale

**Affiliations:** 1 Walter Reed Biosystematics Unit, Smithsonian Institution Museum Support Center, 4210 Silver Hill Road, Suitland, MD 20746, USA Smithsonian Institution Museum Support Center Suitland United States of America; 2 Entomology Branch, Walter Reed Army Institute of Research, 503 Robert Grant Avenue, Silver Spring, MD 20910, USA Entomology Branch, Walter Reed Army Institute of Research Silver Spring United States of America; 3 Department of Entomology, Smithsonian Institution National Museum of Natural History, Washington, DC 20560, USA Smithsonian Institution National Museum of Natural History Washington United States of America; 4 Laboratório de Biodiversidade Entomológica, Instituto Oswaldo Cruz, Fiocruz, Av. Brasil 4365, Rio de Janeiro, RJ, 21040-900, Brazil Instituto Oswaldo Cruz Rio de Janeiro Brazil

**Keywords:** Chagas disease, Latreille, Peru, South America, vector

## Abstract

The taxonomic status of *Triatomadimidiata* (Latreille, 1811) is, by far, the most discussed within Triatominae. Molecular studies have recovered at least three independently evolving lineages in *T.dimidiata* across its range. The original description of *T.dimidiata* (as *Reduviusdimidiatus*) included few taxonomic characters, and no types were assigned. To define and describe the cryptic diversity within *T.dimidiata* sensu lato (s.l.), a neotype must be designated. For this purpose, all 199 specimens identified as *T.dimidiata* from the collections of the Smithsonian Institution – National Museum of Natural History and the American Museum of Natural History, ranging from Peru to Mexico, were studied. Only one specimen (from Tumbes, Peru) matched the combination of characters as listed in the original description, and it is herein formally designated as the neotype for *T.dimidiata*. The neotype is morphologically described and DNA sequences of its whole mitochondrial genome and the nuclear second internal transcribed spacer region (ITS2), commonly used in triatomine molecular systematics studies, are presented and compared to other publicly available sequences of *T.dimidiata* s.l. in GenBank. Our results suggest that *T.dimidiata* sensu stricto (s.s.) is somewhat rare and, therefore, unlikely to serve as a major vector of Chagas disease.

## ﻿Introduction

*Triatomadimidiata* (Latreille, 1811) (Hemiptera, Reduviidae) has long been assumed to be the most widespread species of Triatominae, a reduviid subfamily of Chagas disease vectors. The long-standing discussion about its taxonomic status, however, is consequential of an original description with no type specimens defined and very few characters described by Latreille (1811).

**Figure 1.**
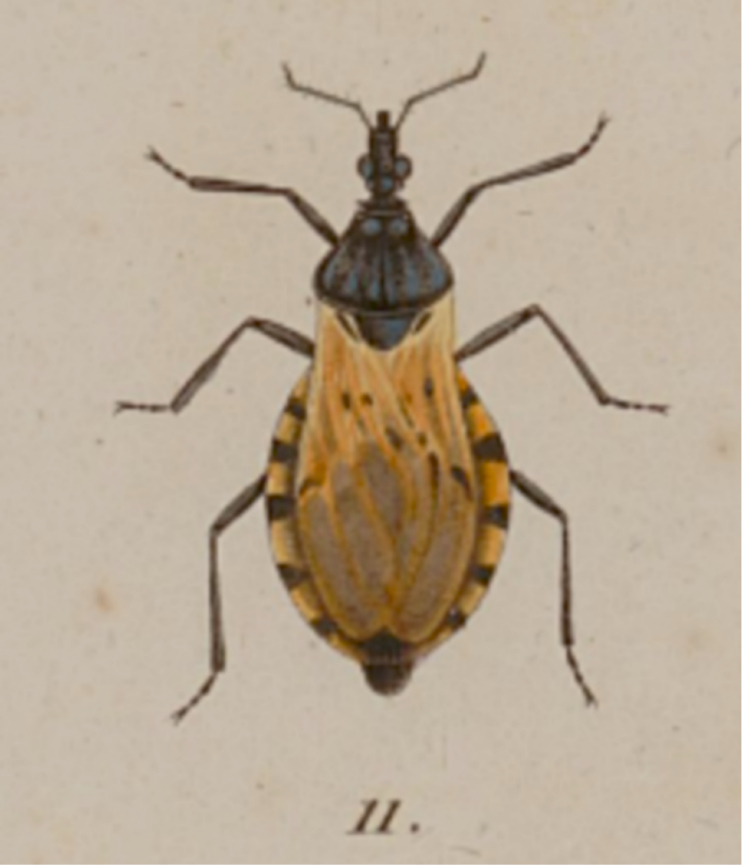
Original drawing presented by Latreille (1811) illustrating the description of *Reduviusdimidiatus*.

*Triatomadimidiata* was originally described as *Reduviusdimidiatus* by Latreille in Humboldt and Bonpland (1811), where the author only provided a drawing of the dorsal view of the described specimen (Fig. 1), a male specimen, pale yellow with body black, and no scale to size. Latreille highlighted the presence of 1+1 discal tubercles and 1+1 lateral tubercles on the pronotum, as well as 1+1 subapical “spines” on fore- and mid-femora. The locality was listed as Villa de Ybara, Peru (now Ecuador).

About 150 years after the description of *T.dimidiata*, two new species had been described, and later synonymized to *T.dimidiata*: *Triatomacapitata* Usinger, 1941 and *Triatomamaculipennis* Pinto, 1931. In their taxonomic revision of Triatominae, Lent and Wygodzinsky (1979), upon observation of 160 specimens from the whole geographic range (Mexico to Peru), highlighted the “highly variable” coloration and other morphological characters of the specimens observed. They concluded, however, that these specimens have not segregated “into clearly separable allopatric populations”; synonimizing both *T.capitata* and *T.maculipennis* with *T.dimidiata*.

The availability of molecular sequencing techniques led to the discovery that *T.dimidiata*, when comprising the recently described *Triatomamopan* Dorn, Justi & Dale, 2018 and *Triatomahuehuetenanguensis* Lima-Cordón & Justi, 2019, is a paraphyletic species complex (Justi et al. 2014b, 2016) which includes at least three independently evolving lineages (Bargues et al. 2008; Dorn et al. 2009, 2016; Justi et al. 2018).

Here we set out to assign and describe a neotype of *T.dimidiata* sensu stricto (s.s.) conforming to the original description of Latreille (1811) and from as close to the original locality as possible, in order to facilitate the understanding of the systematics of *T.dimidiata* sensu lato (s.l.). This solid taxonomic platform allows for the conduct of future studies to facilitate better understanding of the composition and internal systematics of species comprising *Triatomadimidiata* s.l.. Given that most of the diversity in *T.dimidiata* s.l. is reported based on molecular rather than morphological data, the whole mitochondrial genome and nuclear rDNA sequences of the second internal transcribed spacer (ITS2) of the neotype specimen are also presented.

## ﻿Methods

### ﻿Examined material

To locate an optimal neotype in the absence of designated types in the original description of *T.dimidata* (Latreille, 1811), we examined all available specimens of *T.dimidiata* s.l. in the Hemiptera collections of the Smithsonian Institution-National Museum of Natural History (USNM) (*n* = 106) and of the American Museum of Natural History (AMNH) (*n* = 93). Following a first pass, all specimens were further compared to the original description and drawing by Latreille (1811). Where morphological deviations from the original description were observed, the examined specimen was discarded as a potential neotype. Associated metadata for all examined specimens are available as Suppl. material 1.

Once the specimen, suitable to be designated as neotype (i.e., showing no deviation from the characters originally described and collected close to the type locality) was identified, measurements were taken using a Dino-Lite Edge digital microscope. Whole specimen (habitus) and detailed character photos were taken at the NMNH Scanning Electron Microscopy Imaging Lab, using an Olympus DSX100 camera.

### ﻿Mitochondrial genome and ITS-2 sequencing and assembly

Non-destructive DNA extraction, NGS library preparation, as well as mitochondrial genome and ITS2 sequencing and assembly were performed as previously described (Justi et al. 2021). References used for the sequence assemblies included *T.dimidiata* s.l. mitochondrial genome (GenBank accession AF01594) and *T.dimidiata* s.l. haplotype T-dim-H65 internal transcribed spacer 2 (GenBank accession KT874451).

### ﻿Molecular barcode-like analysis

Two alignments were constructed to assess the relatedness of the neotype to the previously studied sequences: (1) all CytB sequences available on GenBank labeled as *T.dimidiata*; and (2) all ITS2 sequences available on GenBank labeled as *T.dimidiata*. Each of these public datasets were combined with the respective sequences from the neotype, *T.mopan*, and *T.huehuetenanguensis* and aligned using the Geneious alignment algorithm implemented in Geneious (Kearse et al. 2012). The GenBank accession number list for the sequences included is available as Suppl. material 2.

Pairwise Kimura 2-parameter (Kimura 1980) distances were calculated for comparison with previously published *T.dimidiata* s.l. sequences using the package ape (Paradis et al. 2004), and barcode-like gap analysis, followed by cluster analysis were performed for both markers, using a custom R script (R Development Core Team 2013). R code is available as Suppl. material 3. Briefly, pairwise distances are calculated for all sequences, and sorted from smallest to largest. The difference between the distances is successively calculated for each value, subtracting the previous value from the current value. The highest difference is identified. Then, the two distances that generated that difference are identified and assigned as the barcode-like gap. The barcode-like gap is then used to identify clusters, using the pairwise distances between the sequences.

## ﻿Results

### ﻿Examined material

**Figure 2.**
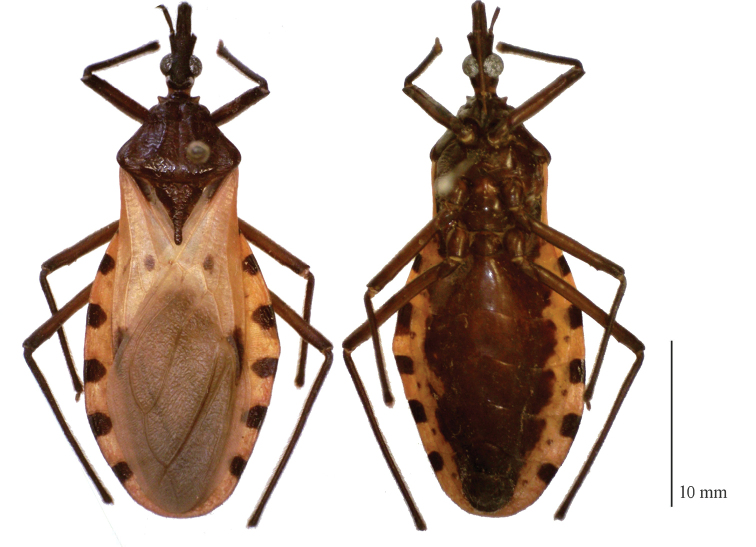
*Triatomadimidiata* neotype. Male.

Among the 199 specimens available in the collections of the AMNH and USNM, there were no exemplars from the original locality of Villa de Ybara (previously Peru; now part of Ecuador). Only one examined specimen presented the character combinations as per the original description of Latreille (1811): 1+1 discal tubercles, 1+1 lateral tubercles and, 1+1 subapical denticles on the femur on the front and mid leg. The male specimen (Fig. 2), from Tumbes, Peru, was previously illustrated in figure 63 in the review by Lent and Wygodzinsky (1979), and is herein designated as the neotype. As this was the only specimen determined to be *T.dimidiata* s.s., the character measurements refer only to the neotype specimen (Table 1).

**Table 1.** Character measurements of the *Triatomadimidiata* neotype.

Character	Length (mm)
Total body length	29.0
Width of the abdomen	11.5
Length of pronotum	5.5
Width of pronotum	7.2
Length of head (excludes neck)	5.0
Width of head across eyes	3.1
Synthlipsis	1.7
Width of eye in dorsal view	1.4
Anteocular region	3.5
Postocular region	1.7
Diameter of ocellus	0.5
Distance between ocellus-eye	0.2
Labium – Length of 1^st^ visible segment	1.0
Labium – Length of 2^nd^ visible segment	1.8
Labium – Length of 3^rd^ visible segment	0.9

### ﻿Mitochondrial genome

**Figure 3.**
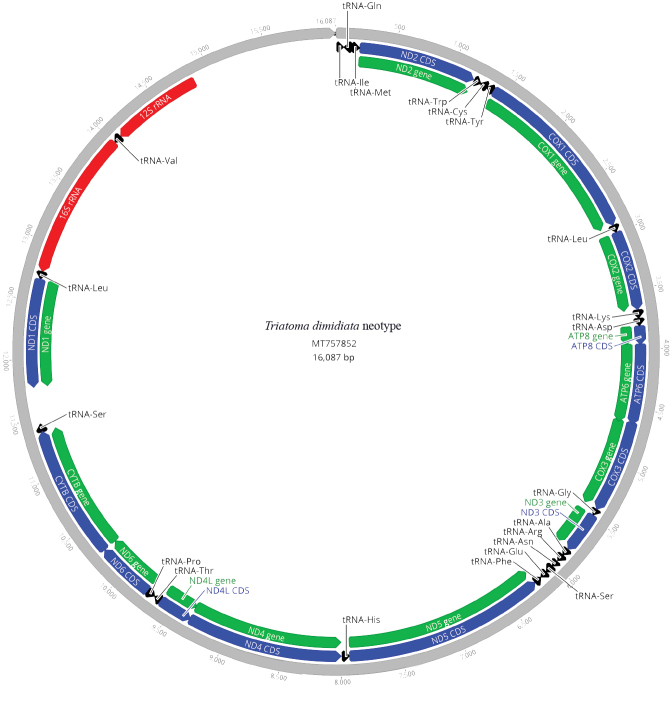
*Triatomadimidiata* neotype (male) mitochondrial genome.

The assembled mitochondrial genome (GenBank MT757852) comprises 16,087 nucleotides, with all 13 typical mitochondrial coding genes, 22 tRNA genes, and 12S and 16S ribosomal regions (Suppl. material 4). These were observed to follow the same arrangement as the reference used (GenBank AF01594; Fig. 3).

### ﻿Molecular barcode-like analysis

Based on the calculated barcode-like gap clustering analysis for the mtDNA CytB alignment created, which comprised 105 sequences, only one sequence, from El Salvador [KT998327; Haplotype T.dim-Cytb.DKin (Dorn et al. 2016)] clustered with the neotype. For this marker, the lowest observed intra-cluster distance was zero, while the highest was 0.00334 (average 0.00115, SD ± 0.0014); while observed inter-cluster distance ranged from 0.00488–0.1754 (average 0.0881, SD ± 0.05446). Calculated CytB distances between the neotype and *T.mopan*, and the neotype and *T.huehuetenanguensis* were 0.1388 and 0.1580, respectively.

This diversity was further echoed through clustering analysis of all publicly available *T.dimidiata* nuclear rDNA ITS2 sequences (*n* = 128). Some 60 clusters were recovered, with 14 sequences, with geographic origin ranging throughout Central America (Table 2), clustering with the neotype.

The lowest observed intra-cluster distance for ITS-2 sequences was zero, while the highest was 0.00464 (average 0.00214, SD ± 0.0019); the lowest observed inter-cluster distance was 0.00674 and the highest 0.15164 (average 0.05998, SD ± 0.0267). Estimated ITS-2 distances between the neotype and *T.mopan*, and the neotype and *T.huehuetenanguensis* were 0.0326 and 0.0399, respectively.

**Figure 4.**
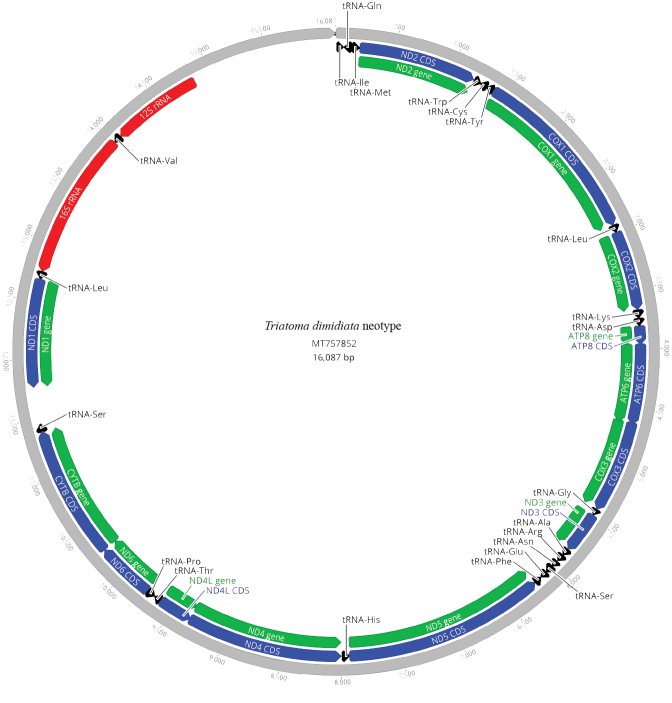
Pairwise Kimura 2-parameter distances between publicly available sequences and sequences from the neotype. Dashed lines indicate the barcode-like gap for each marker.

A graphical representation of the calculated CytB and ITS-2 distances between the neotype and all other sequences is presented on Figure 4.

**Table 2.** List of GenBank accessions and geographic origin of specimens for which the ITS-2 sequence clustered with the neotype sequence.

GenBank species label	GenBank accession	Geographic origin as listed on the original publication for the sequence
*Triatomadimidiata*	AM286696.1	Guatemala: Quiche
*Triatomadimidiata*	DQ871355.1	El Salvador: Santa Ana
*Triatomadimidiata*	AM286697.1	Ecuador: Guayaquil
*Triatomadimidiata*	MN505087.1	Ecuador
*Triatomadimidiata*	MN505088.1	Ecuador
*Triatomadimidiata*	KT874433.1	Costa Rica
*Triatomadimidiata*	KT874432.1	Costa Rica
*Triatomadimidiata*	KF192843.1	Costa Rica
*Triatomadimidiata*	AM286700.1	Guatemala: Pueblo Nuevo
*Triatomadimidiata*	AM286693.1	Guatemala: Jutiapa
*Triatomadimidiata*	AM286701.1	Honduras: Yoro Yoro
*Triatomadimidiata*	AM286694.1	Honduras: Yoro Yoro
*Triatomadimidiata*	MK248260.1	Mexico: Chiapas
*Triatomadimidiata*	MK248261.1	Mexico: Chiapas

## ﻿Description of the neotype

### ﻿Taxonomy

#### Family Reduviidae Latreille, 1807

##### Subfamily Triatominae Jeannel, 1919

###### ﻿Genus Triatoma

Taxon classificationAnimaliaHemipteraReduviidae

Laporte, 1832

FE2900CB-9C31-562A-869F-3C53A637C567

Triatoma dimidiata (Latreille, 1811)
Reduvius dimidiatus Latreille, 1811
Conorhinus dimidiatus (Stål, 1859)
Conorrhinus dimidiatus (Champion, 1899)
Triatoma dimidiata (Neiva, 1914)

####### Type.

***Neotype***: male, designated herein. Cabezza de Lagartos, Tumbes, Peru. [= fig. 63 of Lent and Wygodzinsky (1979)].

**Locality data**: Cabezza de Lagartos, Tumbes, Peru [no date or collector attributes given].

**Depository**: American Museum of Natural History, specimen number AMNH_IZC 00319810.

**DNA**: neotype mitochondrial genome (GenBank MT757852); neotype nuclear ribosomal second internal spacer region (ITS2) (GenBank MT362613).

####### Diagnosis.

The neotype can be immediately distinguished from the other observed *T.dimidiata* s.l. specimens by the following combination of character: pronotum with anterior lobe presenting 1+1 discal tubercles (one on each side of anterior lobe), pointed posteriorly, and 1+1 round, smaller lateral tubercles. Legs uniformly dark brown, one pair of subapical denticles on fore and middle femora; femur and tarsi setose with setae same color as tegument. Spongy fossula observed on fore- and mid-legs.

####### Description.

***Measurements***: Table 1. Coloration: generally brown, with connexivum and wings yellow. Head brown with setosity lighter than the tegument. Labium with first visible segment dark brown and second visible segment slightly paler than first. Neck brown, with 1+1 dark-yellow stripe. Pronotum brown, anterior lobe slightly darker than posterior lobe. Collar brown with anterolateral angles yellowish apically (Fig. 2A, B). Hemelytra with corium and most part of the clavus yellow and membrane smoky-brown. Basal portion of clavus brown, subcostal vein almost all yellow, except apex which bears a brown claw-shaped spot. Small dark oval spots adjacent (above, but not over) m-cu cross vein. Legs uniformly dark with femora and tarsi setosity with same color as tegument. Connexivum in dorsal view mostly yellow, with brown spots on first third of each segment adjacent to sutures (Fig. 2A). In ventral view, abdomen mostly brown on the center, with unique continuous yellow band separating the connexival segments and the center (brown) of the sternites (Fig. 2B).

**Figure 5.**
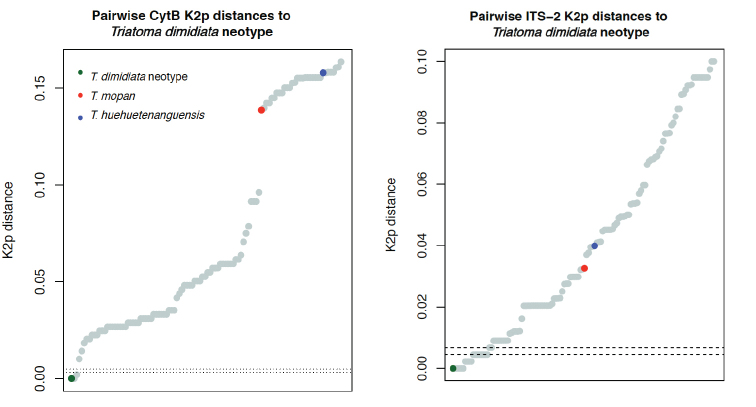
*Triatomadimidiata* neotype head detail (male) **A** dorsal view **B** lateral view.

***Structure***: head shallowly rugose on dorsal view, less than twice as long as width across the eyes (1: 0.62), and slightly shorter than the pronotum (1:1.1). Anteocular region about twice as long as the postocular region (1:0.48) (Fig. 5A). Eyes surpassing the ventral but not the dorsal margin of the head (Fig. 5B), in lateral view. Ratio of the width of eye to synthlipsis: 1:1.21. Ocelli larger than the distance from the eyes to ocelli (1:0.4) and inserted on conspicuous C-shaped protuberance (Fig. 5A).

Antenniferous tubercles subcylindrical, situated slightly after the posterior half of the anteocular region (Fig. 5A). First antennal segment not attaining to level of apex of clypeus; other antennal segments missing (Fig. 5A). Labium slender, with first visible segment not reaching the level of the base of antenniferous tubercle; second visible segment extending to neck; third visible segment reaching the anterior third of stridulatory sulcus (Fig. 5B). Ratio of visible labium segments: 1:1.8:0.9.

**Figure 6.**
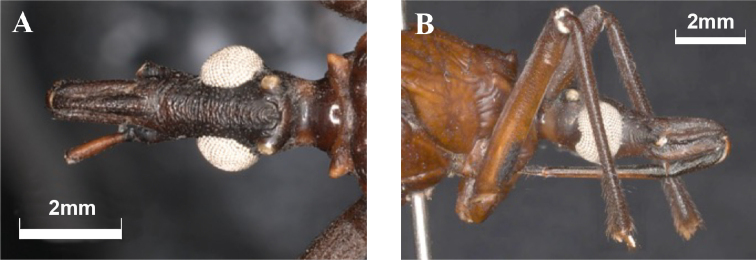
Pronotum of *Triatomadimidiata* neotype (male). Detail of the anterior lobe of the pronotum, showing the discal tubercle (right arrow) and lateral tubercle (left arrow).

**Figure 7.**
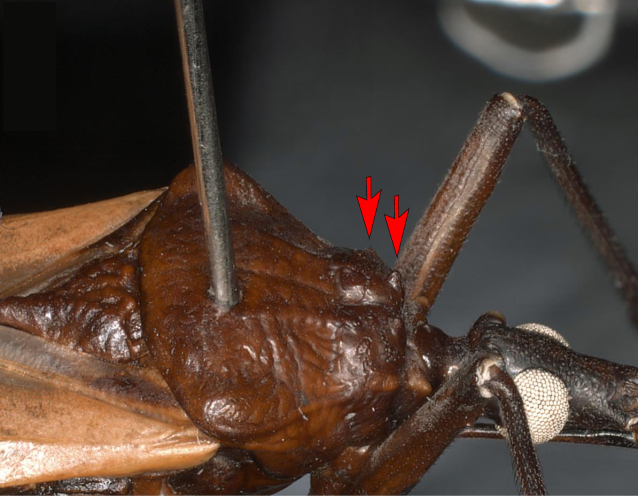
*Triatomadimidiata* neotype (male). Detail of head, pronotum, and scutellum in dorsal view

Pronotum with anterior lobe presenting a distinct depression and with 1+1 discal tubercle, pointed posteriorly, and 1+1 round, smaller lateral tubercles (Fig. 6). Anterolateral angles presenting almost triangular, anterolaterally directed. Posterior lobe slightly rugose. Humeral angles slightly elevated and rounded. Scutellum rugose; posterior process of scutellum with rounded apex, shorter than basal portion of scutellum (Fig. 7).

**Figure 8.**
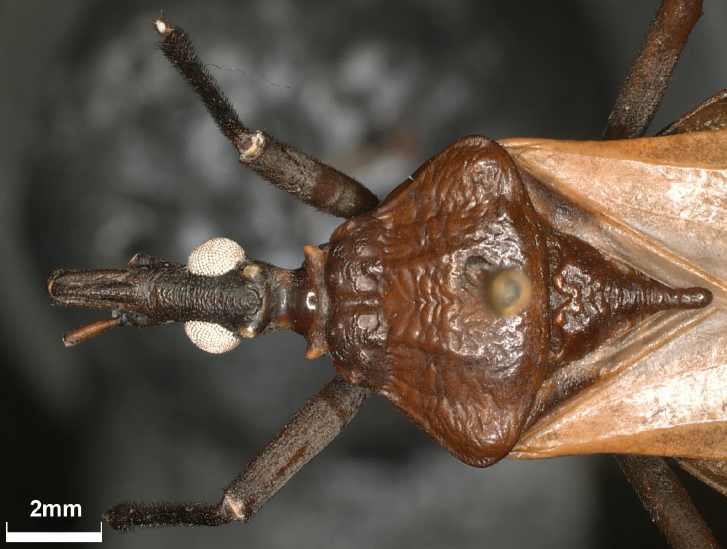
Legs of *Triatomadimidiata* neotype (male) **A** detail of the pair of subapical denticles present on the forelegs **B** pair of subapical denticles present on the midlegs. Hindlegs do not present denticles.

Hemelytra not reaching the posterior margin of VII urotergite (Fig. 2A). Legs with one pair of subapical denticles on fore and mid femora (Fig. 8); spongy fossae on the apices of fore- and mid-tibiae.

Abdomen ventrally convex, delicately striate transversally. Width of abdomen 0.39 times the total body length (1:0.39). Abdominal spiracles adjacent to connexival suture, surrounded by a round brown spot.

## ﻿Discussion

The specific status of *Triatomadimidiata* s.l., a major vector of Chagas disease in the New World, has long been debated among Triatominae systematists (Lent and Wygodzinsky 1979; Dorn et al. 2007, 2016; Bargues et al. 2008; Monteiro et al. 2013; Justi et al. 2018). Two taxa originally described as separate species and later designated as *T.dimidiata* subspecies-*Triatomadimidiatamaculipennis* (Neiva 1914) and *Triatomadimidiatacapitata* Usinger, 194–were synonymized with *T.dimidiata* by Lent and Wygodzinsky (1979). With the application of molecular approaches, it has become increasingly clear that the entity commonly described as *T.dimidiata* comprises several species, which may or may not differ in vector competency. Most recently, two other species-*T.mopan* and *T.huehuetenanguensis*-have been formally described within the *T.dimidiata* group. Adding to the incredible diversity and, at the same time, similarity of the *T.dimidiata* s.l. lineages, is the fact that when a cluster analysis is performed, over 50 distinct clusters are found, regardless of the marker used. However, when compared to other lineages within Triatominae, pairwise distances within *T.dimidiata* s.l. for both CytB and ITS2 are much lower.

The designation of a neotype was critical to fix the identity of *T.dimidiata* s.s. and better understand the component members of this very diverse and epidemiologically important group moving forward. In this study, our goal was to find a specimen as close as possible as the one Latreille described in 1811. With Latreille’s original specimens untraceable, we re-examined all USNM and AMNH*T.dimidiata* specimens used by Lent and Wygodzinky (1979) in their work, searching for the optimal specimen fitting the original description, to assign as the neotype. We noted that, while all exhibit a similar overall morphology, specimens markedly differed in several important taxonomic features from one locality to another, suggesting that the specimens used by Lent and Wygodzinsky (1979) in fact comprised several closely related taxa within *T.dimidiata* s.l.. These findings go some way to explaining the wide diversity of taxonomic characters, bionomics, and distribution currently attributed to *T.dimidiata* in the literature.

The most recent example of how deeper knowledge of *T.dimidiata* s.l. taxonomy is necessary, is the great effort by Rengifo-Correa et al. (2021). In this study, the authors performed a morphological review and published a long overdue diagnostic key to the species of the *T.phyllosoma* species group. Unfortunately, however, the taxonomic uncertainty regarding the status of *T.dimidiata* led the authors to include, as such, what seems to be at least three distinct lineages [see fig. 3D-F of Rengifo-Correa et al. (2021)]. Upon comparison of the diagnosis for *T.dimidiata* provided there, and the neotype, several disparities were found: (1) the anteocular region of the neotype is 2× the length of the postocular region, not 2.5–3× as stated by Rengifo-Correa et al., (2) the first antennal segment does not reach the apex of the clypeus on the neotype, (3) comparison of the images [fig. 3D-F of Rengifo-Correa et al. (2021)] shows disparities in the shape and coloration of the anterolateral angles; shape, color, and rugosity of the pronotum; and shape, color, and location of the wing spots, in relation to the neotype.

Despite examining all 199 available specimens housed in the AMNH and USNM collections, we found only one specimen that exactly matched Latreille’s (1811) original description of *T.dimidata*. Similarly, only one of over 100+ publicly available mtDNA CytB sequences clustered with the neotype. Clustering analyses of ITS-2 sequences, however, showed the neotype sequence to be grouped with 14 other publicly available samples.

These observations are consistent with the hypothesis previously raised that *T.dimidiata* s.s. would have originated in northern Central America and been, somehow, introduced to South America (Bargues et al. 2008). Combined with the young age (<10 My) of the *T.dimidiata* s.l. lineage (Justi et al. 2016), these results show that the radiation of the group likely occurred very quickly and recently, like the radiation of south American *Triatoma* (Justi et al. 2016) that comprise several morphologically differentiable species, but for which current widely used molecular markers cannot be used for reliable species identification (Justi et al. 2014a).

With ITS-2 being a more conserved genetic marker, it is cautious to rely on CytB for the most accurate identification of such a young lineage, which lead us to conclude that true *T.dimidiata* is a rare species, with a seemingly limited range; contradicting current opinion that it is one of the most widespread species of the genus *Triatoma*, and consequently casting doubt on its perceived role as the major vector of Chagas’ disease in the New World. Efforts are urgently needed to better assess the taxonomic status of the genetically diverse entities within the *T.dimidiata* s.l. and assess which of the component taxa are truly involved in transmission of this increasingly important disease in the New World.

## Supplementary Material

XML Treatment for Triatoma

## Supplementary materials

Supplementary material 1 Specimens metadata Data type COL https://binary.pensoft.net/file/620383This dataset is made available under the Open Database License (http://opendatacommons.org/licenses/odbl/1.0/). The Open Database License (ODbL) is a license agreement intended to allow users to freely share, modify, and use this Dataset while maintaining this same freedom for others, provided that the original source and author(s) are credited.Silvia Andrade Justi, Carolina Dale

Supplementary material 2 GenBank numbers list Data type molecular data https://binary.pensoft.net/file/620384This dataset is made available under the Open Database License (http://opendatacommons.org/licenses/odbl/1.0/). The Open Database License (ODbL) is a license agreement intended to allow users to freely share, modify, and use this Dataset while maintaining this same freedom for others, provided that the original source and author(s) are credited.Silvia Andrade Justi, Carolina Dale

Supplementary material 3 R code for barcode-like and cluster analyses 3 Data type statistical data https://binary.pensoft.net/file/620385This dataset is made available under the Open Database License (http://opendatacommons.org/licenses/odbl/1.0/). The Open Database License (ODbL) is a license agreement intended to allow users to freely share, modify, and use this Dataset while maintaining this same freedom for others, provided that the original source and author(s) are credited.Silvia Andrade Justi, Carolina Dale

Supplementary material 4 Mitochondrial genome gene table Data type molecular data https://binary.pensoft.net/file/620386This dataset is made available under the Open Database License (http://opendatacommons.org/licenses/odbl/1.0/). The Open Database License (ODbL) is a license agreement intended to allow users to freely share, modify, and use this Dataset while maintaining this same freedom for others, provided that the original source and author(s) are credited.Silvia Andrade Justi, Carolina Dale
