# Supplementary material for: ﻿Designation of the neotype of Triatomadimidiata (Latreille, 1811) (Hemiptera, Reduviidae, Triatominae), with full integrated redescription including mitogenome and nuclear ITS-2 sequences
Source: Zookeys. 2021 Dec 8;1076:9–24. doi: 10.3897/zookeys.1076.72835 (PMC8674215; doi:10.3897/zookeys.1076.72835)
Supplement: Supplementary material 2 — GenBank numbers list [file zookeys-1076-009-s002.docx]

**GenBank access number for the sequences used in the barcode-like analyses, besides the neotype sequences**

**Cytochrome B**

*Triatoma huehuetenanguensis*: MG951754

*Triatoma mopan*: MG954249

*Triatoma dimidiata:* AF301594; AY062149; AY062150; AY062151; AY062152; AY062153; AY062154; AY062155; AY062156; AY062157; AY062158; AY062159; AY062160; AY062161; AY062162; AY062163; AY062164; AY859417; AY859418; FJ197154; FJ197155; FJ197156; FJ197157; FJ197158; FJ197159; FN641804; FN641805; FN641806; FN641807; FN641808; FN641809; FN641810; FN641811; FN641812; FN641813; FN641814; FN641815; FN641816; FN641817; FN641818; KP775964; KP775965; KP775967; KP775968; KP775969; KP775970; KP775971; KP775972; KP775973; KP775974; KP775975; KP775976; KP775977; KP775978; KT998289; KT998290; KT998291; KT998292; KT998293; KT998294; KT998295; KT998296; KT998297; KT998298; KT998299; KT998300; KT998301; KT998302; KT998303; KT998304; KT998305; KT998306; KT998307; KT998308; KT998309; KT998310; KT998311; KT998312; KT998313; KT998314; KT998315; KT998316; KT998317; KT998318; KT998319; KT998320; KT998321; KT998322; KT998323; KT998324; KT998325; KT998326; KT998327; KT998328; KT998329; KT998330; KT998331; KT998332; MT556656; MT556657; MT556666; NC_002609.

**ITS-2**

*Triatoma huehuetenanguensis*: MG947605

*Triatoma mopan*: MG954252

*Triatoma dimidiata*: AJ286875; AJ286876; AJ286877; AJ286878; AJ286879; AJ286880; AM286693; AM286694; AM286695; AM286696; AM286697; AM286698; AM286699; AM286700; AM286701; AM286702; AM286703; AM286704; AM286705; AM286706; AM286707; AM286708; AM286709; AM286710; AM286711; AM286712; AM286713; AM286714; AM286715; AM286716; AM286717; AM286718; AM286719; AM286720; AM286721; AM286722; AM286723; AY860408; AY860409; AY860410; AY860411; AY860412; AY860413; AY860414; AY860415; AY860416; AY860417; DQ871354; DQ871355; DQ871356; EF383122; EF383123; EF383124; EF383125; EF383126; EF383127; EF383128; EF383129; FJ197146; FJ197147; FJ197148; FJ197149; FJ197150; FJ197151; FJ197152; FJ197153; GQ214508; GQ214509; GQ214510; GQ214511; GQ214512; GQ214513; KC489292; KC489293; KC489294; KC489295; KC489296; KC489297; KC489298; KC489299; KC489300; KC489301; KC489302; KC489303; KC489304; KC489305; KC489306; KC489307; KC489308; KF192843; KF192844; KF192845; KF192846; KF192847; KT321467; KT321468; KT321469; KT321470; KT874431; KT874432; KT874433; KT874434; KT874435; KT874436; KT874437; KT874438; KT874439; KT874440; KT874441; KT874442; KT874443; KT874444; KT874445; KT874446; KT874447; KT874448; KT874449; KT874450; KT874451; M505086; M505087; M505088; M505089; MK248260; MK248261;
