# Supplementary material for: ﻿Designation of the neotype of Triatomadimidiata (Latreille, 1811) (Hemiptera, Reduviidae, Triatominae), with full integrated redescription including mitogenome and nuclear ITS-2 sequences
Source: Zookeys. 2021 Dec 8;1076:9–24. doi: 10.3897/zookeys.1076.72835 (PMC8674215; doi:10.3897/zookeys.1076.72835)
Supplement: Supplementary material 3 — R code for barcode-like and cluster analyses 3 [file zookeys-1076-009-s003.pdf]

# Triatoma dimidiata neotype - CytB cluster analysis

Silvia A. Justi

6/10/2021

#Libraries used

```
library(ape)
library(dplyr)
library(reshape2)
library(tidyverse)
library(data.table)
library(plyr)
```

#Read CytB alignment alignment and calculate pairwise distances

```
data <- read.FASTA("CytB_all.fasta")
dist_data <- as.data.frame(dist.dna(data, model = "K80",
                    as.matrix = TRUE, pairwise.deletion = T))
d<-dist_data #this "d" will be used later
write.csv(dist_data, file="dist_data.csv") #save pairwise distances to file
```

#Format the table to a 3 column pairwise comparison, and keep only query sample name and calculated distance value.

```
df<-reshape2::melt(dist_data)
```

#Sort the pairwise distance values select only the values between 0.01 and 0.05. Because 0.00 should mean intraspecific distance and 0.05 is usually interspecific, for this marker for insects.

```
sorted_dists<-as.data.table(df[order(df$value, decreasing = FALSE),])
dists_filtered<-sorted_dists[sorted_dists$value <= 0.05 ]
dists_filtered2<-dists_filtered[dists_filtered$value > 0.00]
```

#Take a quick look ate the values filtered for the intra-inter specific distances region.

```
plot(dists_filtered2$value)
```

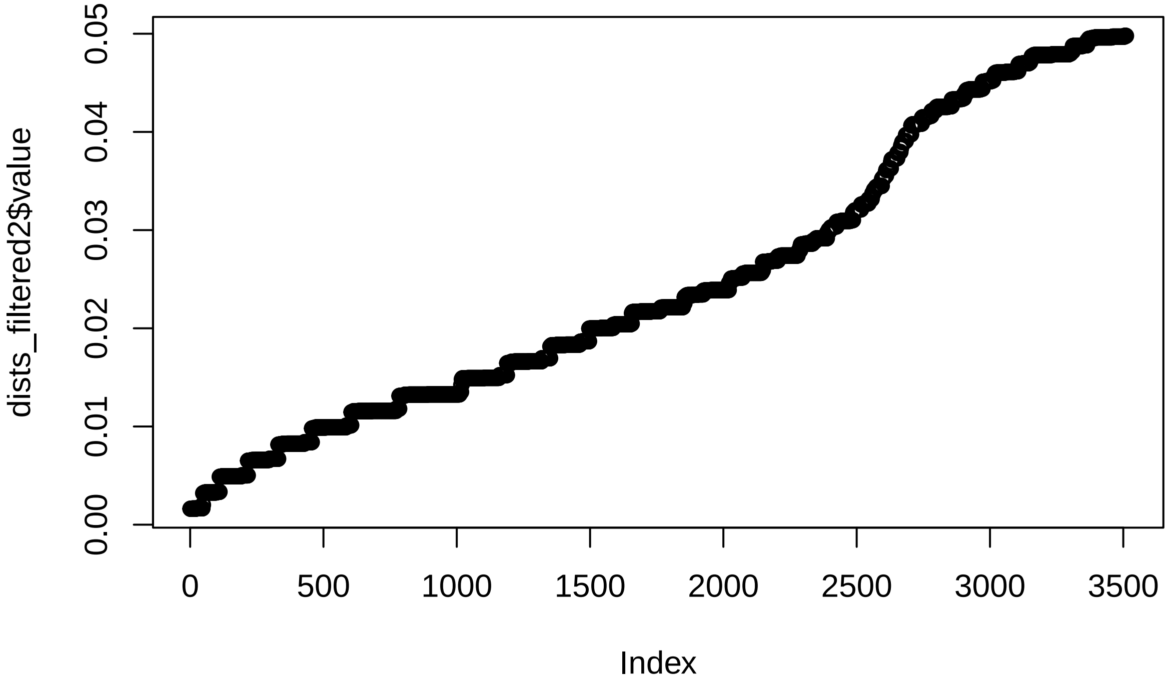


#Now find the first barcode-like gap, by subtracting the distance value, by the previous distance value successively

```
dt <- as.data.table(dists_filtered2)
setkey(dt, value)
dt[, diff := value - shift(value, fill = first(value))]
```

#And plot the differences to take a look

```
plot(sort(dt$diff))
```

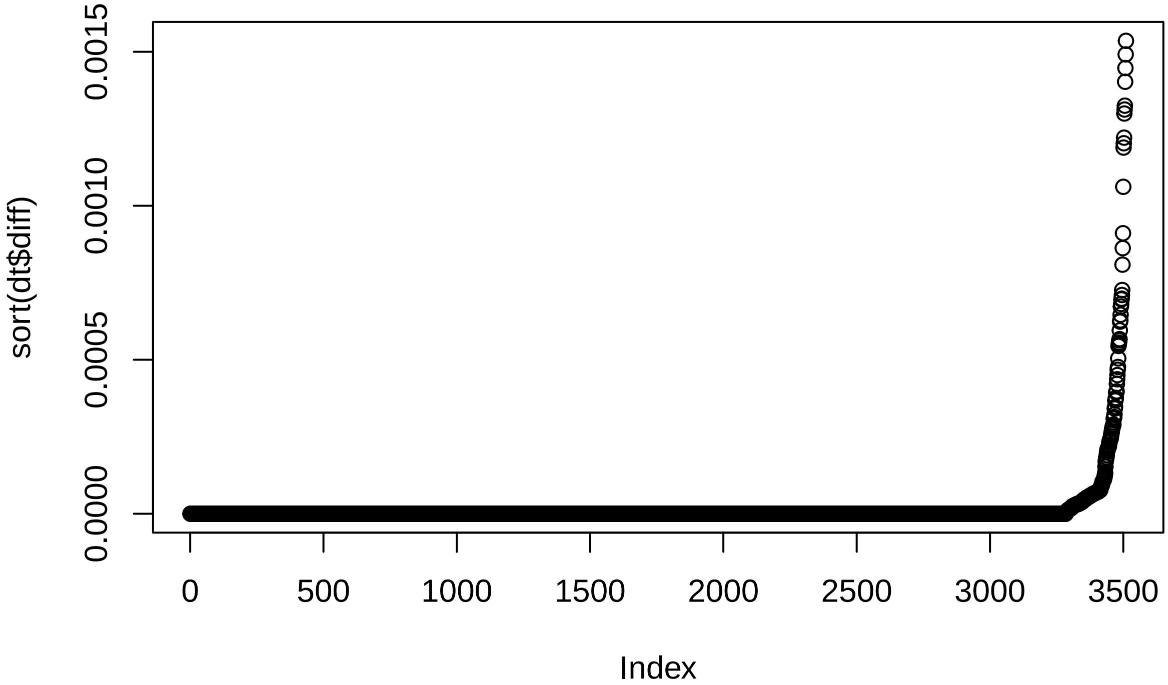

#Check the aproximate distance of the first observed gap, and round that number down. E.g. if the first observed gap is around y=0.0015, use 0.001

```
dt_high<-dt[dt$diff > 0.00050 ]
dt_high #look at the values
```

```
##                                      variable       value         diff
##  1:             Triatoma_dimidiata_FJ197157.1 0.003205139 0.0011890074
##  2:             Triatoma_dimidiata_AY062155.1 0.004880060 0.0015355656
##  3:             Triatoma_dimidiata_FJ197159.1 0.006516722 0.0014915545
##  4:             Triatoma_dimidiata_AY062164.1 0.008158760 0.0014472498
##  5:             Triatoma_dimidiata_AY062164.1 0.009806208 0.0014026488
##  6:             Triatoma_dimidiata_AY062164.1 0.011459102 0.0013251207
##  7:             Triatoma_dimidiata_FJ197158.1 0.013117478 0.0013125455
##  8:             Triatoma_dimidiata_FN641805.1 0.014240781 0.0007264444
##  9:             Triatoma_dimidiata_FJ197156.1 0.014803713 0.0005629323
## 10:             Triatoma_dimidiata_FJ197156.1 0.016450824 0.0012212203
## 11:             Triatoma_dimidiata_MT556666.1 0.018153809 0.0012030333
## 12:             Triatoma_dimidiata_KT998327.1 0.019977430 0.0012995365
## 13:             Triatoma_dimidiata_AY062155.1 0.021526503 0.0010615759
## 14:             Triatoma_dimidiata_AY062153.1 0.023184933 0.0006279519
## 15:             Triatoma_dimidiata_KT998324.1 0.024593652 0.0006983198
## 16:            Triatoma_dimidiata_NC_002609.1 0.026767532 0.0008624837
## 17:             Triatoma_dimidiata_AY062164.1 0.029722920 0.0005551313
## 18:             Triatoma_dimidiata_FJ197158.1 0.031822679 0.0008088369
## 19:             Triatoma_dimidiata_KT998327.1 0.032609949 0.0005043277
## 20:             Triatoma_dimidiata_FJ197155.1 0.035192371 0.0006959349
## 21:             Triatoma_dimidiata_FN641812.1 0.036116959 0.0005669868
## 22:             Triatoma_dimidiata_FJ197154.1 0.036931503 0.0006461564
## 23:             Triatoma_dimidiata_FN641812.1 0.037852191 0.0005447477
## 24:             Triatoma_dimidiata_KT998314.1 0.039694792 0.0006236783
## 25: Triatoma_dimidiata_KP775976.1_(reversed) 0.040663768 0.0009108408
## 26:             Triatoma_dimidiata_FN641818.1 0.041436436 0.0005954089
## 27:             Triatoma_dimidiata_KT998316.1 0.043298411 0.0006811833
## 28:             Triatoma_dimidiata_KT998315.1 0.045110007 0.0007102464
## 29:             Triatoma_dimidiata_FN641810.1 0.046861444 0.0006727723
## 30:             Triatoma_dimidiata_KT998316.1 0.048753010 0.0005498226
##                                      variable       value         diff
```

#Now, find the max difference, which will be the correspondent to higher end of the barcode gap

```
max(dt_high$diff)
```

```
## [1] 0.001535566
```

#Now, find the samples that generated the gap

```
t<-dt$diff < max(dt_high$diff)
min(which(t == FALSE))
```

```
## [1] 111
```

```r
row_id<-(min(which(t == FALSE)))
dt[(row_id-1):(row_id+1),]
```

```
##                           variable       value        diff
## 1: Triatoma_dimidiata_FN641804.1 0.003344494 0.000000000
## 2: Triatoma_dimidiata_AY062155.1 0.004880060 0.001535566
## 3: Triatoma_dimidiata_AY062153.1 0.004880060 0.000000000
```

#Find the gap using the values calculated above

```r
intra<-as.matrix(dist_data)
inter<-as.matrix(dist_data)
suppressMessages(intra[intra>min((dt[(row_id-1):(row_id+1),]) %>% select (2))] <- NA)
suppressMessages(inter[inter<max((dt[(row_id-1):(row_id+1),]) %>% select (2))] <- NA)
suppressMessages(inter[inter>=1]<- NA)
df_final <- data.frame(melt(intra), melt(inter)) %>% select(1, 3,6)
names(df_final) <- c("lineage", "intra_ID", "inter_ID")
```

#Plot the gap with all samples

```r
boxplot(df_final$inter_ID ~ df_final$lineage,  ylim=c(0,0.15),
        ylab = "K2p pairwise distance", border= "gray31", xaxt = "n", xlab = "",
        col="gray47", pch=21, frame=F, cex=0.5)

boxplot(df_final$intra_ID ~ df_final$lineage, add=TRUE, ylim=c(0,0.15),
        ylab = "K2p pairwise distance", border= "gray61", xaxt = "n", xlab = "",
        col="gray90", pch=21, frame=F, cex=0.5)
abline(h=min((dt[(row_id-1):(row_id+1),]) %>% select (2)), col="green",lty=3)
abline(h=max ((dt[(row_id-1):(row_id+1),]) %>% select (2)), col="red",lty=3)
```

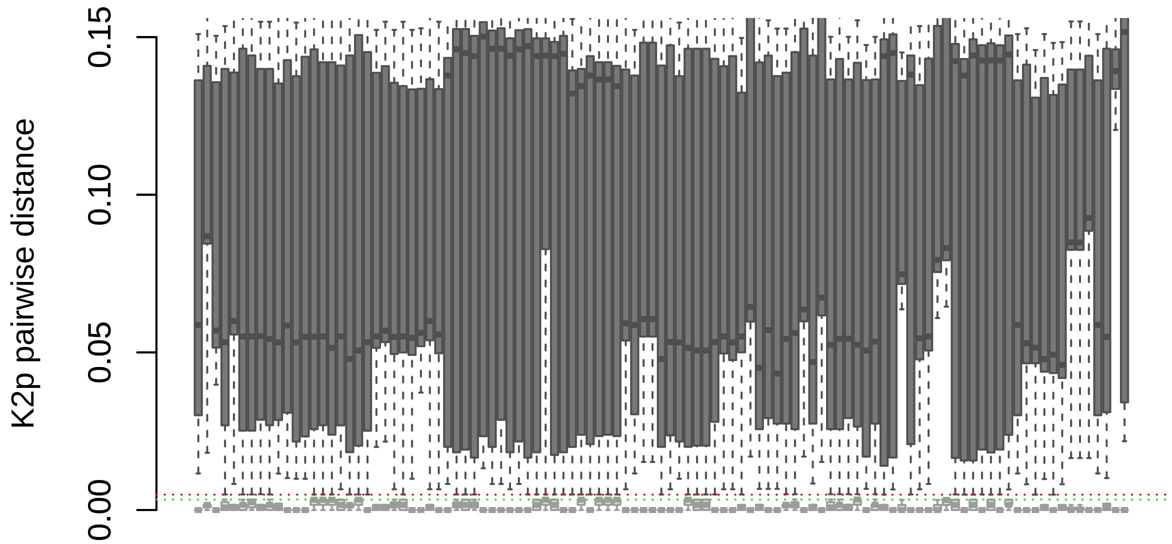


#And the barcode gap is…

```r
min((dt[(row_id-1):(row_id+1),]) %>% select (2))
```

```
## [1] 0.003344494
```

```
max ((dt[(row_id-1):(row_id+1),]) %>% select (2))
```

```
## [1] 0.00488006
```

#Write the files with the comparisons and find the lowest, highest, mean and standard devidation of the intraspecific and interspecific distances

```
groups<-reshape2::melt(intra)
#write.csv(groups, file="intra_groups.csv")
min(df_final$intra_ID, na.rm = TRUE)
```

```
## [1] 0
```

```
max(df_final$intra_ID, na.rm = TRUE)
```

```
## [1] 0.003344494
```

```
mean(df_final$intra_ID, na.rm = TRUE)
```

```
## [1] 0.001149609
```

```
sd(df_final$intra_ID, na.rm = TRUE)
```

```
## [1] 0.001394133
```

```
groups_inter<-reshape2::melt(inter)
#write.csv(groups_inter, file="inter_groups.csv")
min(df_final$inter_ID, na.rm = TRUE)
```

```
## [1] 0.00488006
```

```
max(df_final$inter_ID, na.rm = TRUE)
```

```
## [1] 0.1753962
```

```
mean(df_final$inter_ID, na.rm = TRUE)
```

```
## [1] 0.08818151
```

```
sd(df_final$inter_ID, na.rm = TRUE)
```

```
## [1] 0.05446892
```

#Write the groups and plot the clusters

```
dist_data<-as.dist(dist_data)
hc<-hclust(dist_data,"complete")
plot(hclust(dist_data),cex=0.3)
abline(h= round(min((dt[(row_id-1):(row_id+1),]) %>%
                      select (2)),digits=7), col="red",lty=3)
```

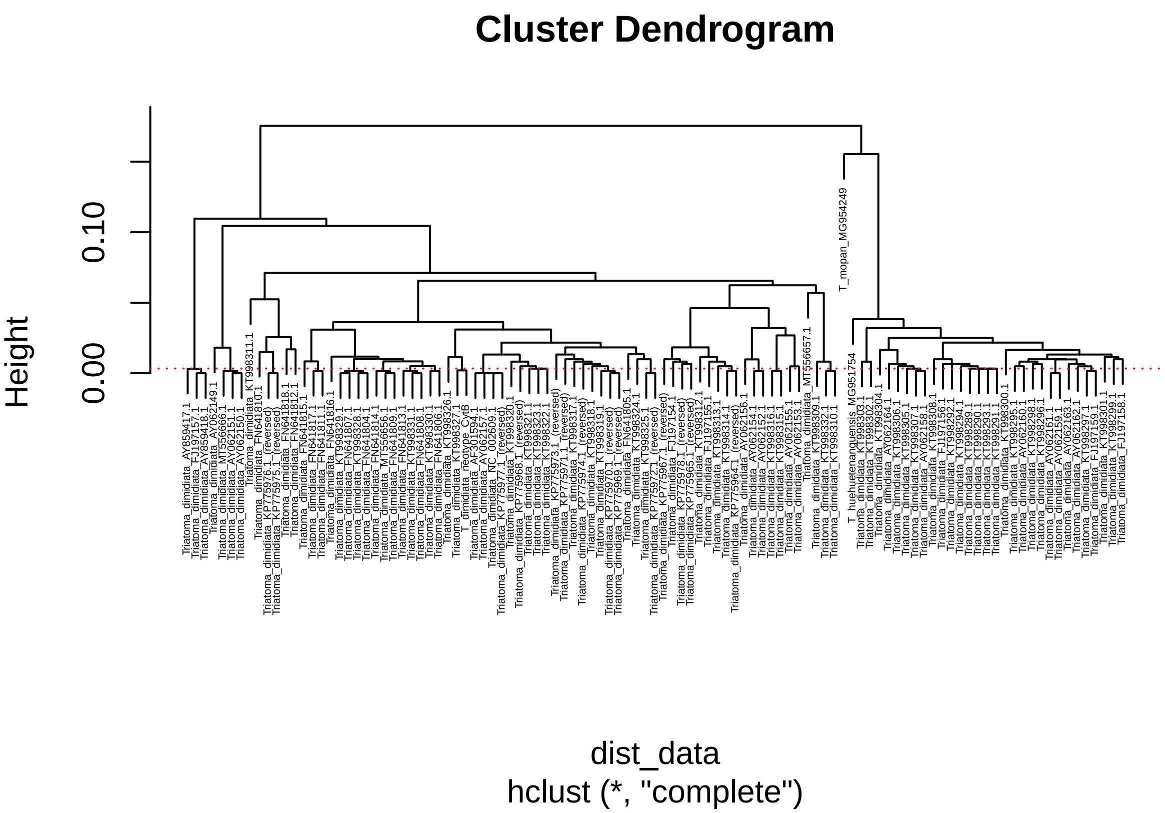


```
final_clusters<-as.data.frame(cutree(hc,
              h =  round(min((dt[(row_id-1):(row_id+1),])
               %>% select(2)),digits=7)))
names(final_clusters) <- c("lineage")
final_clusters[order(final_clusters$lineage), , drop = FALSE]
```

```
##                                           lineage
## Triatoma_dimidiata_NC_002609.1                  1
## Triatoma_dimidiata_KP775977.1_(reversed)        1
## Triatoma_dimidiata_AY062157.1                   1
## Triatoma_dimidiata_AF301594.1                   1
## Triatoma_dimidiata_MT556666.1                   2
## Triatoma_dimidiata_AY062151.1                   2
## Triatoma_dimidiata_AY062150.1                   2
## Triatoma_dimidiata_MT556657.1                   3
## Triatoma_dimidiata_MT556656.1                   4
## Triatoma_dimidiata_FN641814.1                   4
## Triatoma_dimidiata_FN641809.1                   4
## Triatoma_dimidiata_KT998332.1                   5
## Triatoma_dimidiata_KT998310.1                   5
## Triatoma_dimidiata_KT998331.1                   6
```

```
## Triatoma_dimidiata_FN641813.1                  6
## Triatoma_dimidiata_FN641808.1                  6
## Triatoma_dimidiata_KT998330.1                  7
## Triatoma_dimidiata_FN641806.1                  7
## Triatoma_dimidiata_KT998329.1                  8
## Triatoma_dimidiata_KT998328.1                  8
## Triatoma_dimidiata_FN641807.1                  8
## Triatoma_dimidiata_FN641804.1                  8
## Triatoma_dimidiata_KT998327.1                  9
## T_dimidiata_neotype_CytB                       9
## Triatoma_dimidiata_KT998326.1                 10
## Triatoma_dimidiata_KT998325.1                 11
## Triatoma_dimidiata_KP775972.1_(reversed)      11
## Triatoma_dimidiata_KT998324.1                 12
## Triatoma_dimidiata_KT998323.1                 13
## Triatoma_dimidiata_KT998322.1                 13
## Triatoma_dimidiata_KT998321.1                 13
## Triatoma_dimidiata_KT998320.1                 14
## Triatoma_dimidiata_KT998319.1                 15
## Triatoma_dimidiata_KP775970.1_(reversed)      15
## Triatoma_dimidiata_KP775969.1_(reversed)      15
## Triatoma_dimidiata_KT998318.1                 16
## Triatoma_dimidiata_KT998317.1                 17
## Triatoma_dimidiata_KT998316.1                 18
## Triatoma_dimidiata_KT998315.1                 18
## Triatoma_dimidiata_KT998314.1                 19
## Triatoma_dimidiata_KP775964.1_(reversed)      19
## Triatoma_dimidiata_KT998313.1                 20
## Triatoma_dimidiata_KT998312.1                 21
## Triatoma_dimidiata_KT998311.1                 22
## Triatoma_dimidiata_KT998309.1                 23
## Triatoma_dimidiata_KT998308.1                 24
## Triatoma_dimidiata_KT998307.1                 25
## Triatoma_dimidiata_KT998305.1                 25
## Triatoma_dimidiata_AY062158.1                 25
## Triatoma_dimidiata_KT998306.1                 26
## Triatoma_dimidiata_KT998304.1                 27
## Triatoma_dimidiata_KT998303.1                 28
## Triatoma_dimidiata_KT998302.1                 29
## Triatoma_dimidiata_KT998301.1                 30
## Triatoma_dimidiata_KT998300.1                 31
## Triatoma_dimidiata_KT998299.1                 32
## Triatoma_dimidiata_KT998298.1                 33
## Triatoma_dimidiata_KT998297.1                 34
## Triatoma_dimidiata_FJ197159.1                 34
## Triatoma_dimidiata_KT998296.1                 35
## Triatoma_dimidiata_KT998295.1                 36
## Triatoma_dimidiata_KT998294.1                 37
## Triatoma_dimidiata_KT998293.1                 38
## Triatoma_dimidiata_KT998291.1                 38
## Triatoma_dimidiata_KT998290.1                 38
## Triatoma_dimidiata_KT998289.1                 38
## Triatoma_dimidiata_KT998292.1                 39
## Triatoma_dimidiata_KP775978.1_(reversed)      40
```

```
## Triatoma_dimidiata_KP775976.1_(reversed)      41
## Triatoma_dimidiata_KP775975.1_(reversed)      41
## Triatoma_dimidiata_KP775974.1_(reversed)      42
## Triatoma_dimidiata_KP775973.1_(reversed)      43
## Triatoma_dimidiata_KP775971.1_(reversed)      44
## Triatoma_dimidiata_KP775968.1_(reversed)      45
## Triatoma_dimidiata_KP775967.1_(reversed)      46
## Triatoma_dimidiata_KP775965.1_(reversed)      47
## Triatoma_dimidiata_FN641818.1                 48
## Triatoma_dimidiata_FN641817.1                 49
## Triatoma_dimidiata_FN641811.1                 49
## Triatoma_dimidiata_FN641816.1                 50
## Triatoma_dimidiata_FN641815.1                 51
## Triatoma_dimidiata_FN641812.1                 52
## Triatoma_dimidiata_FN641810.1                 53
## Triatoma_dimidiata_FN641805.1                 54
## Triatoma_dimidiata_FJ197158.1                 55
## Triatoma_dimidiata_FJ197157.1                 56
## Triatoma_dimidiata_AY859418.1                 56
## Triatoma_dimidiata_AY859417.1                 56
## Triatoma_dimidiata_FJ197156.1                 57
## Triatoma_dimidiata_FJ197155.1                 58
## Triatoma_dimidiata_FJ197154.1                 59
## Triatoma_dimidiata_AY062164.1                 60
## Triatoma_dimidiata_AY062163.1                 61
## Triatoma_dimidiata_AY062162.1                 62
## Triatoma_dimidiata_AY062161.1                 63
## Triatoma_dimidiata_AY062159.1                 63
## Triatoma_dimidiata_AY062160.1                 64
## Triatoma_dimidiata_AY062156.1                 65
## Triatoma_dimidiata_AY062155.1                 66
## Triatoma_dimidiata_AY062154.1                 67
## Triatoma_dimidiata_AY062152.1                 67
## Triatoma_dimidiata_AY062153.1                 68
## Triatoma_dimidiata_AY062149.1                 69
## T_mopan_MG954249                              70
## T_huehuetenanguensis_MG951754                 71
```

```r
#write.csv(final_clusters,file="final_clusters.csv")
```

```r
dist_data <- dist.dna(data, model = "K80",
                      as.matrix = TRUE, pairwise.deletion = T)
Tdimss<-subset(dist_data, select = ("T_dimidiata_neotype_CytB"))

dist_data <- dist.dna(data, model = "K80",
                      as.matrix = TRUE, pairwise.deletion = T)
Tdimss<-subset(dist_data, select = ("T_dimidiata_neotype_CytB"))
df<-as.data.frame(Tdimss)
Tdimss_sorted<-  as.matrix(df[order(df$T_dimidiata_neotype_CytB), , drop = FALSE])
plot (Tdimss_sorted, col ="azure3", pch=20, cex=1.5,
      main = "Pairwise CytB K2p distances to Triatoma dimidiata neotype",
      xaxt="n", xlab = "" , ylab="K2p distance")
points(grep ("T_huehuetenanguensis_MG951754",
             rownames(Tdimss_sorted)),
```

```
       Tdimss[grep ("T_huehuetenanguensis_MG951754",
                    rownames(Tdimss)),1], col="blue",pch=20, cex=1.5)
points(grep ("T_mopan_MG954249",
             rownames(Tdimss_sorted)),
       Tdimss[grep ("T_mopan_MG954249",rownames(Tdimss)),1], col="red",pch=20, cex=1.5)
points(0, 0, col="darkgreen",pch=20, cex=1.5)
legend(0, 0.17, legend=c("T. dimidiata neotype", "T. mopan", "T. huehuetenanguensis"),
       col=c("darkgreen","red", "blue"), pch =20, bty = "n")
abline(h=min((dt[(row_id-1):(row_id+1),]) %>% select (2)), col="black",lty=2, cex=0.5)
abline(h=max ((dt[(row_id-1):(row_id+1),]) %>% select (2)), col="black",lty=2, cex=0.5)
```

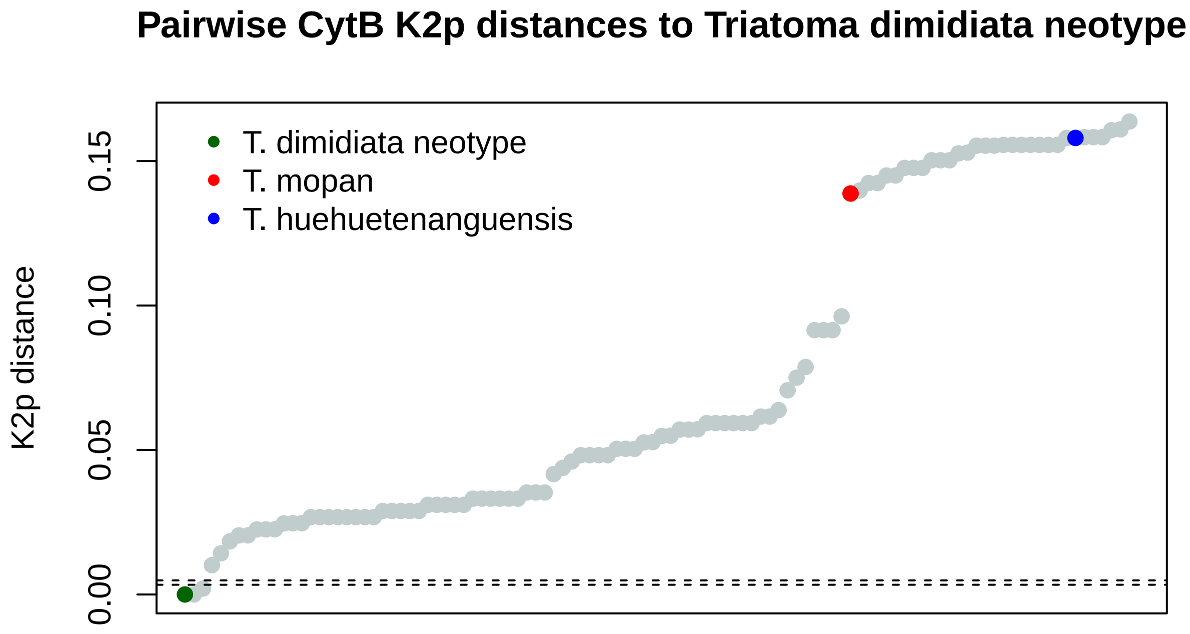

# Triatoma dimidiata neotype - ITS-2 cluster analysis

Silvia A. Justi

6/10/2021

#Libraries used

```
library(ape)
library(dplyr)
library(reshape2)
library(tidyverse)
library(data.table)
library(plyr)
```

#Read ITS2 alignment alignment and calculate pairwise distances

```
data <- read.FASTA("All_dimi_ITS2_alignment.fst")
dist_data <- as.data.frame(dist.dna(data, model = "K80",
                    as.matrix = TRUE, pairwise.deletion = T))
d<-dist_data #this "d" will be used later
write.csv(dist_data, file="dist_data.csv") #save pairwise distances to file
```

#Format the table to a 3 column pairwise comparison, and keep only query sample name and calculated distance value.

```
df<-reshape2::melt(dist_data)
```

#Sort the pairwise distance values select only the values between 0.01 and 0.05. Because 0.00 should mean intraspecific distance and 0.05 is usually interspecific, for this marker for insects.

```
sorted_dists<-as.data.table(df[order(df$value, decreasing = FALSE),])
dists_filtered<-sorted_dists[sorted_dists$value <= 0.05 ]
dists_filtered2<-dists_filtered[dists_filtered$value > 0.00]
```

#Take a quick look ate the values filtered for the intra-inter specific distances region.

```
plot(dists_filtered2$value)
```

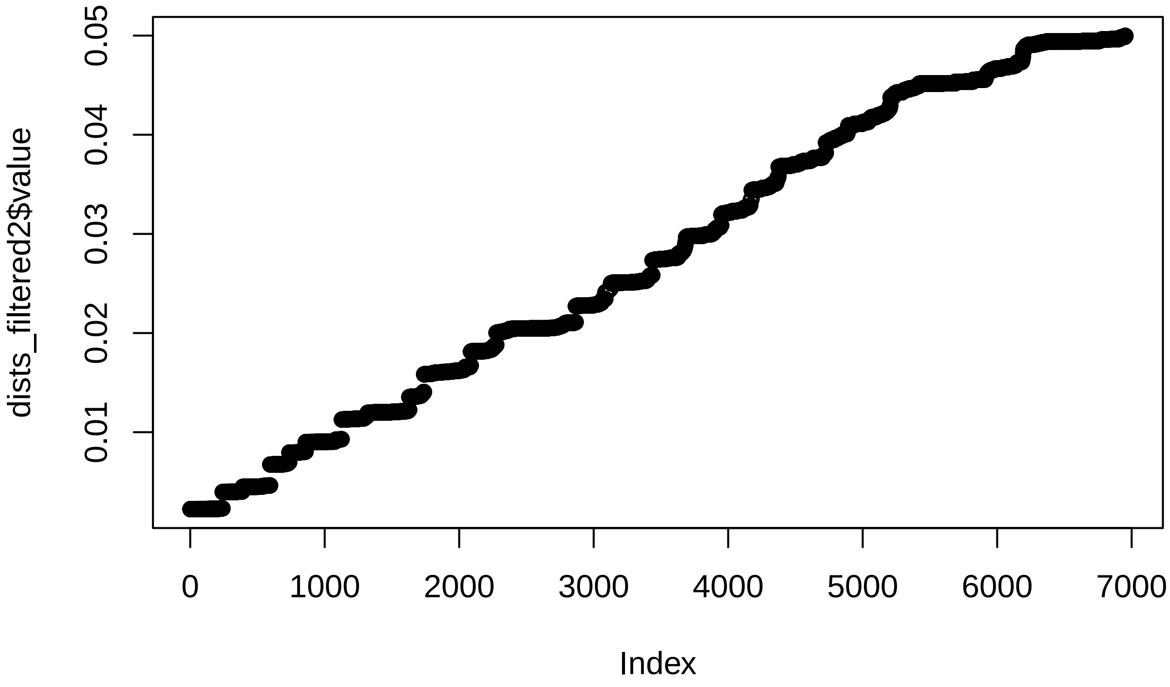


#Now find the first barcode-like gap, by subtracting the distance value, by the previous distance value successively

```
dt <- as.data.table(dists_filtered2)
setkey(dt, value)
dt[, diff := value - shift(value, fill = first(value))]
```

#And plot the differences to take a look

```
plot(sort(dt$diff))
```

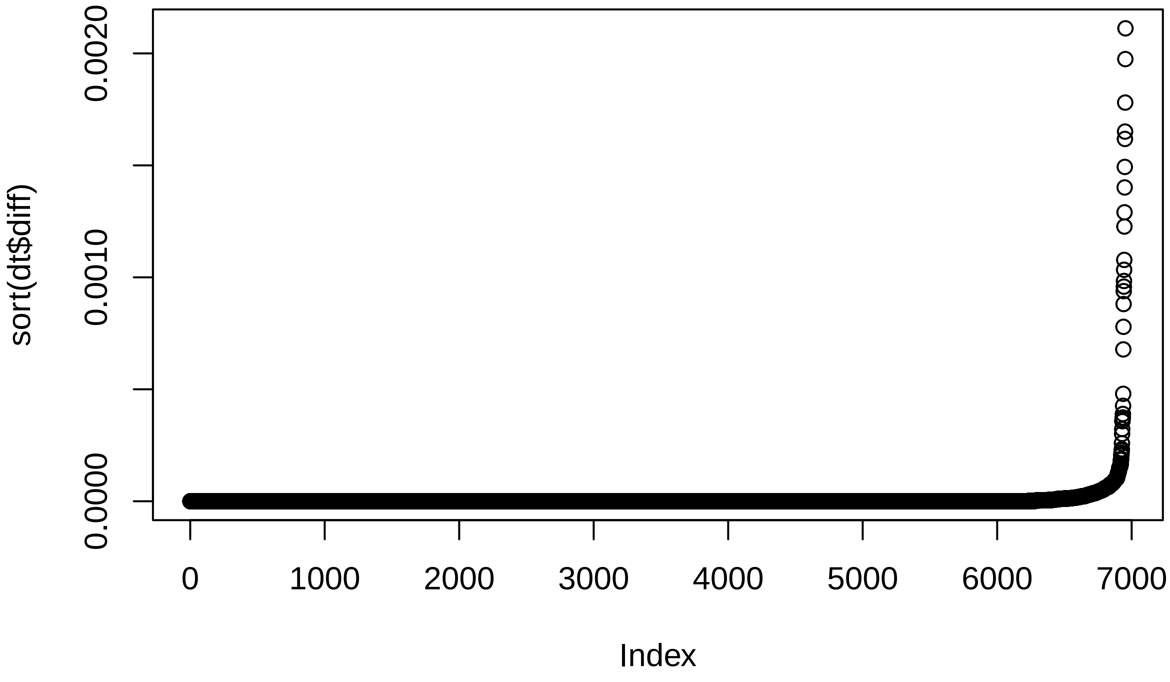

#Check the aproximate distance of the first observed gap, and round that number down. E.g. if the first observed gap is around y=0.0015, use 0.001

```
dt_high<-dt[dt$diff > 0.00030 ]
#dt_high #look at the values
```

#Now, find the max difference, which will be the correspondent to higher end of the barcode gap

```
max(dt_high$diff)
```

```
## [1] 0.002111956
```

#Now, find the samples that generated the gap

```
t<-dt$diff < max(dt_high$diff)
min(which(t == FALSE))
```

```
## [1] 595
```

```
row_id<-(min(which(t == FALSE)))
dt[(row_id-1):(row_id+1),]
```

```
##                          variable       value        diff
## 1: Triatoma_dimidiata_AM286701.1 0.004629663 0.000000000
## 2: Triatoma_dimidiata_KT874431.1 0.006741618 0.002111956
## 3: Triatoma_dimidiata_KT874437.1 0.006741618 0.000000000
```

#Find the gap using the values calculated above

```
intra<-as.matrix(dist_data)
inter<-as.matrix(dist_data)
suppressMessages(intra[intra>min((dt[(row_id-1):(row_id+1),]) %>% select (2))] <- NA)
suppressMessages(inter[inter<max((dt[(row_id-1):(row_id+1),]) %>% select (2))] <- NA)
suppressMessages(inter[inter>=0.05]<- NA)
df_final <- data.frame(melt(intra), melt(inter)) %>% select(1, 3,6)
names(df_final) <- c("lineage", "intra_ID", "inter_ID")
```

#Plot the gap with all samples

```
boxplot(df_final$inter_ID ~ df_final$lineage , ylim=c(0,0.06),
        ylab = "K2p pairwise distance", border= "gray61", xaxt = "n", xlab = "",
        col="gray90", pch=21, frame=F, cex=0.5)

boxplot(df_final$intra_ID  ~ df_final$lineage , add=TRUE, ylim=c(0,0.06),
        ylab = "K2p pairwise distance", border= "gray61", xaxt = "n", xlab = "",
        col="gray90", pch=21, frame=F, cex=0.5)
abline(h=min((dt[(row_id-1):(row_id+1),]) %>% select (2)), col="green",lty=3)
abline(h=max ((dt[(row_id-1):(row_id+1),]) %>% select (2)), col="red",lty=3)
```

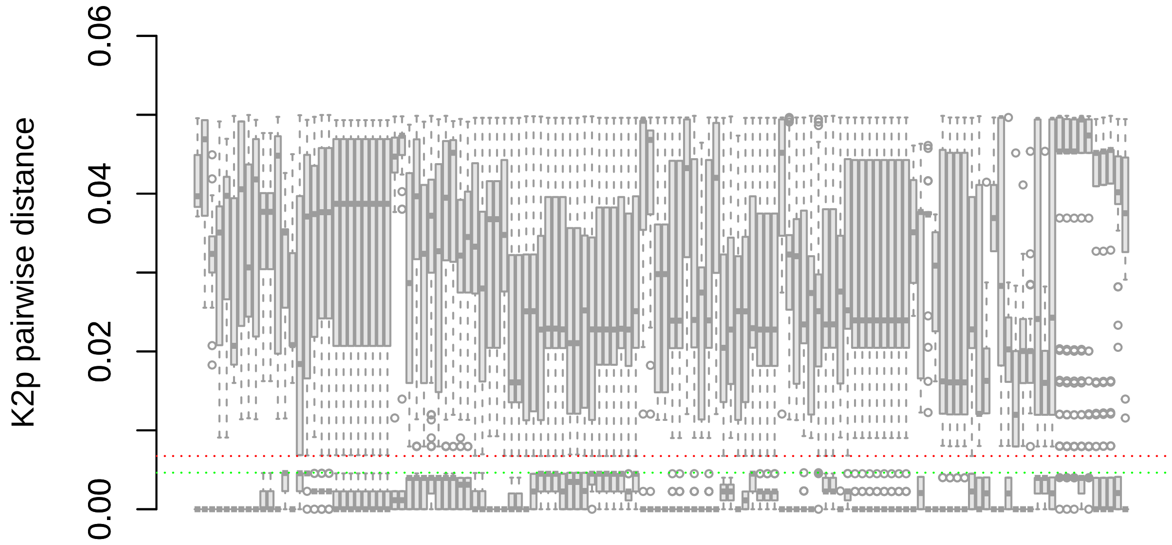


#And the barcode gap is…

```
min((dt[(row_id-1):(row_id+1),]) %>% select (2))
```

```
## [1] 0.004629663
```

```
max ((dt[(row_id-1):(row_id+1),]) %>% select (2))
```

```
## [1] 0.006741618
```

#Write the files with the comparisons and find the lowest, highest, mean and standard devidation of the intraspecific and interspecific distances

```
groups<-reshape2::melt(intra)
#write.csv(groups, file="intra_groups.csv")
min(df_final$intra_ID, na.rm = TRUE)
```

```
## [1] 0
```

```
max(df_final$intra_ID, na.rm = TRUE)
```

```
## [1] 0.004629663
```

```
mean(df_final$intra_ID, na.rm = TRUE)
```

```
## [1] 0.001634742
```

```
sd(df_final$intra_ID, na.rm = TRUE)
```

```
## [1] 0.001870817
```

```r
groups_inter<-reshape2::melt(inter)
#write.csv(groups_inter, file="inter_groups.csv")
min(df_final$inter_ID, na.rm = TRUE)
```

```
## [1] 0.006741618
```

```r
max(df_final$inter_ID, na.rm = TRUE)
```

```
## [1] 0.04997256
```

```r
mean(df_final$inter_ID, na.rm = TRUE)
```

```
## [1] 0.03005757
```

```r
sd(df_final$inter_ID, na.rm = TRUE)
```

```
## [1] 0.0138779
```

#Write the groups and plot the clusters

```r
dist_data<-as.dist(dist_data)
hc<-hclust(dist_data,"complete")
plot(hclust(dist_data),cex=0.3)
abline(h= round(min((dt[(row_id-1):(row_id+1),]) %>%
                      select (2)),digits=7), col="red",lty=3)
```

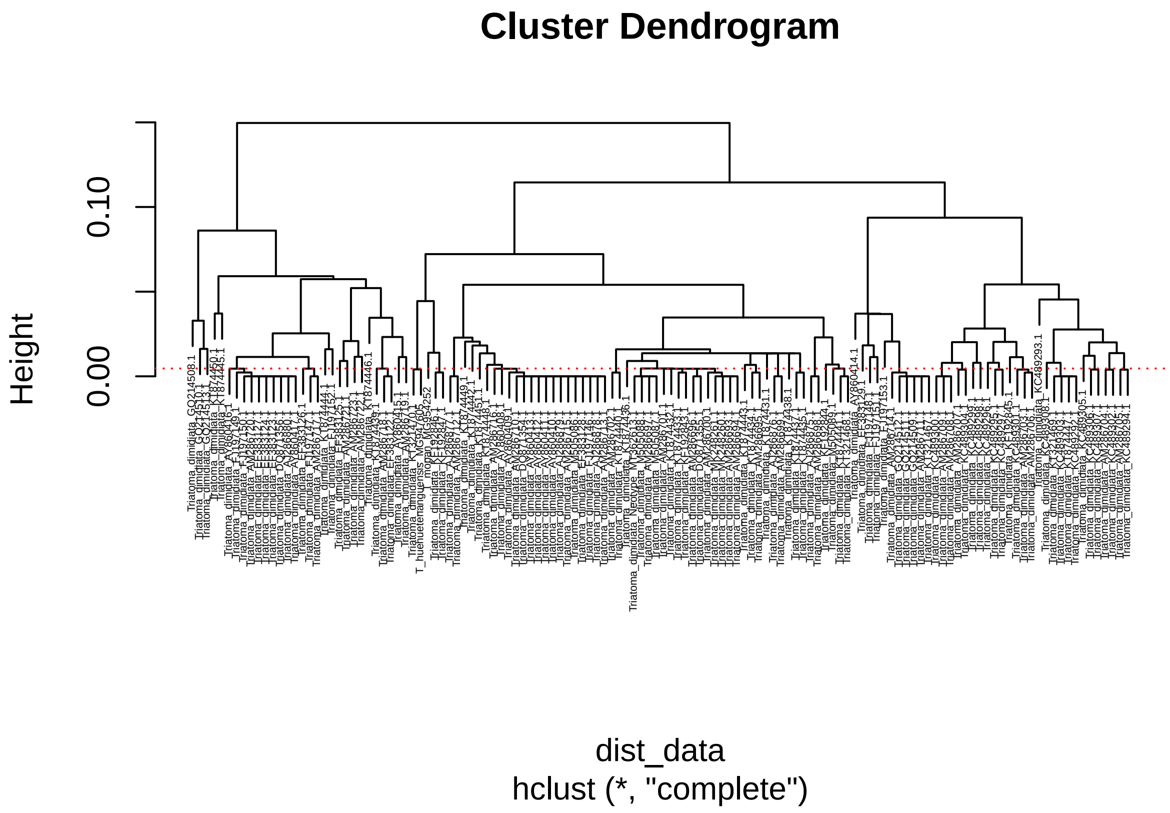

```r
final_clusters<-as.data.frame(cutree(hc,
                h =  round(min((dt[(row_id-1):(row_id+1),]) %>% select(2)),digits=7)))
names(final_clusters) <- c("lineage")
final_clusters[order(final_clusters$lineage), , drop = FALSE]
```

```
##                                      lineage
## Triatoma_dimidiata_KT874450.1              1
## Triatoma_dimidiata_KT874445.1              2
## Triatoma_dimidiata_KT874446.1              3
## Triatoma_dimidiata_EF383125.1              4
## Triatoma_dimidiata_AM286721.1              5
## Triatoma_dimidiata_KT874444.1              6
## Triatoma_dimidiata_AY860415.1              7
## Triatoma_dimidiata_AM286723.1              8
## Triatoma_dimidiata_AM286719.1              9
## Triatoma_dimidiata_AM286718.1             10
## Triatoma_dimidiata_EF383122.1             10
## Triatoma_dimidiata_KT874439.1             10
## Triatoma_dimidiata_AM286722.1             11
## Triatoma_dimidiata_FJ197152.1             12
## Triatoma_dimidiata_EF383126.1             13
## Triatoma_dimidiata_FJ197147.1             13
## Triatoma_dimidiata_AM286717.1             13
## Triatoma_dimidiata_AY860416.1             14
## Triatoma_dimidiata_FJ197149.1             14
## Triatoma_dimidiata_AJ286880.1             14
## Triatoma_dimidiata_AY860417.1             14
## Triatoma_dimidiata_DQ871356.1             14
## Triatoma_dimidiata_EF383123.1             14
## Triatoma_dimidiata_EF383124.1             14
## Triatoma_dimidiata_EF383127.1             14
## Triatoma_dimidiata_AM286720.1             14
## Triatoma_dimidiata_FJ197150.1             14
## Triatoma_dimidiata_KF192846.1             15
## Triatoma_dimidiata_KF192847.1             15
## Triatoma_dimidiata_KT874447.1             16
## Triatoma_dimidiata_KC489292.1             16
## Triatoma_dimidiata_KC489303.1             16
## Triatoma_dimidiata_KT321469.1             16
## Triatoma_dimidiata_AM286709.1             17
## Triatoma_dimidiata_AM286708.1             17
## Triatoma_dimidiata_KC489300.1             17
## Triatoma_dimidiata_AM286704.1             18
## Triatoma_dimidiata_KC489302.1             18
## Triatoma_dimidiata_KF192845.1             19
## Triatoma_dimidiata_AM286705.1             20
## Triatoma_dimidiata_KC489294.1             20
## Triatoma_dimidiata_AM286707.1             21
## Triatoma_dimidiata_KC489304.1             21
## Triatoma_dimidiata_AM286703.1             22
## Triatoma_dimidiata_AM286706.1             22
## Triatoma_dimidiata_KC489301.1             22
## Triatoma_dimidiata_KT874441.1             23
```

```
## Triatoma_dimidiata_M505089.1                23
## Triatoma_dimidiata_KT321468.1               23
## Triatoma_dimidiata_AJ286875.1               24
## Triatoma_dimidiata_AM286698.1               24
## Triatoma_dimidiata_KF192844.1               25
## Triatoma_dimidiata_AJ286876.1               26
## Triatoma_dimidiata_AM286699.1               26
## Triatoma_dimidiata_KT874431.1               27
## Triatoma_dimidiata_KT874443.1               28
## Triatoma_dimidiata_KT874437.1               29
## Triatoma_dimidiata_KT874435.1               29
## Triatoma_dimidiata_AM286696.1               30
## Triatoma_dimidiata_DQ871355.1               30
## Triatoma_dimidiata_AM286697.1               30
## Triatoma_dimidiata_M505087.1                30
## Triatoma_dimidiata_M505088.1                30
## Triatoma_dimidiata_KT874433.1               30
## Triatoma_dimidiata_KT874432.1               30
## Triatoma_dimidiata_KF192843.1               30
## Triatoma_dimidiata_AM286700.1               30
## Triatoma_dimidiata_AM286693.1               30
## Triatoma_dimidiata_AM286701.1               30
## Triatoma_dimidiata_AM286694.1               30
## Triatoma_dimidiata_MK248260.1               30
## Triatoma_dimidiata_MK248261.1               30
## Triatoma_dimidiata_Neotype_MT362613.1       30
## Triatoma_dimidiata_KT874436.1               31
## Triatoma_dimidiata_KT874434.1               32
## Triatoma_dimidiata_AM286695.1               32
## Triatoma_dimidiata_AM286711.1               33
## Triatoma_dimidiata_AM286714.1               33
## Triatoma_dimidiata_KT321467.1               33
## Triatoma_dimidiata_GQ214509.1               33
## Triatoma_dimidiata_GQ214511.1               33
## Triatoma_dimidiata_GQ214512.1               33
## Triatoma_dimidiata_AJ286877.1               34
## Triatoma_dimidiata_AM286712.1               34
## Triatoma_dimidiata_AJ286878.1               35
## Triatoma_dimidiata_AM286713.1               35
## Triatoma_dimidiata_FJ197146.1               35
## Triatoma_dimidiata_EF383128.1               35
## Triatoma_dimidiata_M505086.1                35
## Triatoma_dimidiata_AY860409.1               35
## Triatoma_dimidiata_AY860408.1               35
## Triatoma_dimidiata_AM286716.1               35
## Triatoma_dimidiata_AM286715.1               35
## Triatoma_dimidiata_AJ286879.1               35
## Triatoma_dimidiata_AY860410.1               35
## Triatoma_dimidiata_AY860411.1               35
## Triatoma_dimidiata_AY860412.1               35
## Triatoma_dimidiata_AY860413.1               35
## Triatoma_dimidiata_DQ871354.1               35
## Triatoma_dimidiata_AM286710.1               35
## Triatoma_dimidiata_FJ197153.1               36
```

```
## Triatoma_dimidiata_KT874442.1              37
## Triatoma_dimidiata_EF383129.1              38
## Triatoma_dimidiata_AM286702.1              39
## Triatoma_dimidiata_KT874440.1              39
## Triatoma_dimidiata_KT874438.1              40
## Triatoma_dimidiata_AY860414.1              41
## Triatoma_dimidiata_FJ197148.1              42
## Triatoma_dimidiata_KT874449.1              43
## Triatoma_dimidiata_KT874451.1              44
## Triatoma_dimidiata_KT874448.1              45
## Triatoma_dimidiata_FJ197151.1              46
## Triatoma_dimidiata_GQ214508.1              47
## Triatoma_dimidiata_KT321470.1              48
## T_huehuetenanguensis_MG947605              48
## Triatoma_dimidiata_GQ214510.1              49
## Triatoma_dimidiata_GQ214513.1              50
## Triatoma_dimidiata_KC489306.1              51
## Triatoma_dimidiata_KC489307.1              51
## Triatoma_dimidiata_KC489293.1              52
## Triatoma_dimidiata_KC489299.1              53
## Triatoma_dimidiata_KC489296.1              54
## Triatoma_dimidiata_KC489308.1              55
## Triatoma_dimidiata_KC489298.1              56
## Triatoma_dimidiata_KC489305.1              57
## Triatoma_dimidiata_KC489295.1              58
## Triatoma_dimidiata_KC489297.1              58
## T_mopan_MG954252                           59
```

```
#write.csv(final_clusters,file="final_clusters.csv")
```

```
dist_data <- dist.dna(data, model = "K80",
                      as.matrix = TRUE, pairwise.deletion = T)
Tdimss<-subset(dist_data, select = ("Triatoma_dimidiata_Neotype_MT362613.1"))
df<-as.data.frame(Tdimss)
Tdimss_sorted<-  as.matrix(df[order(df$Triatoma_dimidiata_Neotype_MT362613.1), ,
                              drop = FALSE])
plot (Tdimss_sorted, col ="azure3", pch=20, cex=1.5,
      main = "Pairwise ITS-2 K2p distances to Triatoma dimidiata neotype",
      xaxt="n", xlab = "" , ylab="K2p distance")
points(grep ("T_huehuetenanguensis_MG947605",rownames(Tdimss_sorted)),
       Tdimss[grep ("T_huehuetenanguensis_MG947605",rownames(Tdimss)),1],
       col="blue",pch=20, cex=1.5)
points(grep ("T_mopan_MG954252",rownames(Tdimss_sorted)),
       Tdimss[grep ("T_mopan_MG954252",rownames(Tdimss)),1], col="red",
       pch=20, cex=1.5)
points(0, 0, col="darkgreen",pch=20, cex=1.5)
legend(0, 0.1, legend=c("T. dimidiata neotype", "T. mopan",
                        "T. huehuetenanguensis"),
       col=c("darkgreen","red", "blue"), pch =20, bty = "n")
abline(h=min((dt[(row_id-1):(row_id+1),]) %>% select (2)),
       col="black",lty=2, cex=0.5)
abline(h=max ((dt[(row_id-1):(row_id+1),]) %>% select (2)),
       col="black",lty=2, cex=0.5)
```

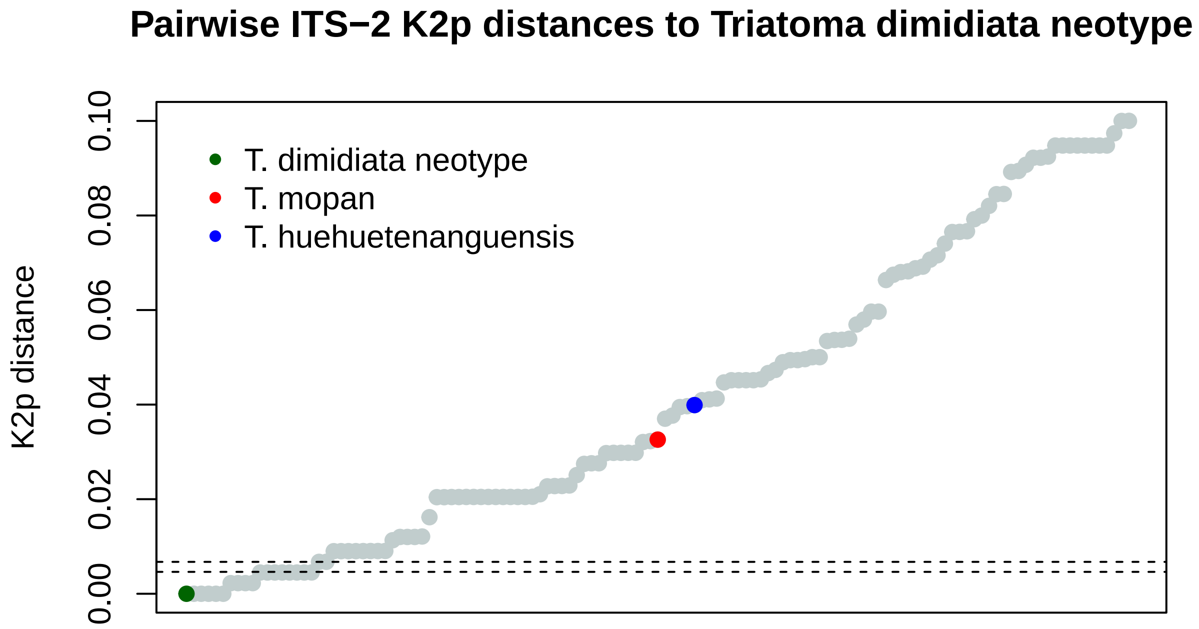
Pairwise ITS−2 K2p distances to Triatoma dimidiata neotype
K2p distance
0.00
0.02
0.04
0.06
0.08
0.10
T. dimidiata neotype
T. mopan
T. huehuetenanguensis
