# Supplementary material for: ﻿Designation of the neotype of Triatomadimidiata (Latreille, 1811) (Hemiptera, Reduviidae, Triatominae), with full integrated redescription including mitogenome and nuclear ITS-2 sequences
Source: Zookeys. 2021 Dec 8;1076:9–24. doi: 10.3897/zookeys.1076.72835 (PMC8674215; doi:10.3897/zookeys.1076.72835)
Supplement: Supplementary material 4 — Mitochondrial genome gene table [file zookeys-1076-009-s004.pdf]

Name	Type	Minimum	Maximum	Length	Direction
type: Neotype sourc...	source	1	16,087	16,087	forward
tRNA-Ile	tRNA	4	67	64	forward
tRNA-Gln	tRNA	65	133	69	reverse
tRNA-Met	tRNA	133	200	68	forward
ND2 gene	gene	201	1,199	999	forward
ND2 CDS	CDS	201	1,199	999	forward
tRNA-Trp	tRNA	1,205	1,271	67	forward
tRNA-Cys	tRNA	1,263	1,326	64	reverse
tRNA-Tyr	tRNA	1,326	1,390	65	reverse
COX1 gene	gene	1,392	2,925	1,534	forward
COX1 CDS	CDS	1,392	2,925	1,534	forward
tRNA-Leu	tRNA	2,925	2,994	70	forward
COX2 gene	gene	2,994	3,672	679	forward
COX2 CDS	CDS	2,994	3,672	679	forward
tRNA-Lys	tRNA	3,673	3,742	70	forward
tRNA-Asp	tRNA	3,742	3,805	64	forward
ATP8 gene	gene	3,806	3,964	159	forward
ATP8 CDS	CDS	3,806	3,964	159	forward
ATP6 gene	gene	3,958	4,641	684	forward
ATP6 CDS	CDS	3,958	4,641	684	forward
COX3 gene	gene	4,628	5,413	786	forward
COX3 CDS	CDS	4,628	5,413	786	forward
tRNA-Gly	tRNA	5,413	5,475	63	forward
ND3 gene	gene	5,476	5,829	354	forward
ND3 CDS	CDS	5,476	5,829	354	forward
tRNA-Ala	tRNA	5,829	5,893	65	forward
tRNA-Arg	tRNA	5,898	5,961	64	forward
tRNA-Asn	tRNA	5,963	6,026	64	forward
tRNA-Ser	tRNA	6,026	6,094	69	forward
tRNA-Glu	tRNA	6,094	6,157	64	forward
tRNA-Phe	tRNA	6,157	6,221	65	reverse
ND5 gene	gene	6,221	7,933	1,713	reverse
ND5 CDS	CDS	6,221	7,933	1,713	reverse
tRNA-His	tRNA	7,934	7,997	64	reverse
ND4 gene	gene	8,000	9,331	1,332	reverse
ND4 CDS	CDS	8,000	9,331	1,332	reverse
ND4L gene	gene	9,325	9,618	294	reverse
ND4L CDS	CDS	9,325	9,618	294	reverse
tRNA-Thr	tRNA	9,621	9,684	64	forward
tRNA-Pro	tRNA	9,685	9,752	68	reverse
ND6 gene	gene	9,753	10,256	504	forward
ND6 CDS	CDS	9,753	10,256	504	forward
CYTB gene	gene	10,256	11,389	1,134	forward
CYTB CDS	CDS	10,256	11,389	1,134	forward
tRNA-Ser	tRNA	11,388	11,456	69	forward
ND1 gene	gene	11,765	12,682	918	reverse
ND1 CDS	CDS	11,765	12,682	918	reverse
tRNA-Leu	tRNA	12,683	12,747	65	reverse
16S rRNA	rRNA	12,748	14,005	1,258	reverse
tRNA-Val	tRNA	14,006	14,075	70	reverse
12S rRNA	rRNA	14,076	14,855	780	reverse
